# The Joint Action on Tobacco Control: A cooperation project for strengthening tobacco control in Europe

**DOI:** 10.18332/tpc/151050

**Published:** 2022-07-01

**Authors:** Mike S. Straarup, Frances O’Donovan, Angeliki Lambrou, Christine Weber, Irina Gebetsberger-Hartleitner, Renata Solimini, Benoît Labarbe, Carl C. Lange, Stine Stærmose, Yvonne C. M. Staal, Anne Havermans, Esteve Fernandez, Dolors Carnicer-Pont, Olena Tigova, Hanna Ollila

**Affiliations:** 1 Danish Safety Technology Authority (DSTA), Esbjerg, Denmark; 2 National Public Health Organization (NPHO), Athens, Greece; 3 Austrian Agency for Health and Food Safety (AGES), Vienna, Austria; 4 Istituto Superiore di Sanità (ISS), National Centre on Addiction and Doping, Rome, Italy; 5 ANSES, Risk Assessment Department, Maisons-Alfort, France; 6 Danish Safety Technology Authority (DSTA), Esbjerg, Denmark; 7 Centre for Health Protection, National Institute for Public Health and the Environment (RIVM), Bilthoven, The Netherlands; 8 Tobacco Control Unit, Catalan Institute of Oncology (ICO); Tobacco Control Research Group, Institut d’Investigació Biomèdica de Bellvitge (IDIBELL); L’Hospitalet, Spain; CIBER Respiratory Diseases (CIBERES), Madrid, Spain; 9 Finnish Institute for Health and Welfare (THL), Helsinki, Finland

**Keywords:** tobacco control, Europe, joint action

Tobacco consumption is the single largest avoidable health risk, and the most significant cause of premature death in the EU, responsible for nearly 0.7 million deaths every year. Around 50% of smokers die prematurely (on average 14 years earlier). Despite considerable progress made in recent years, the number of smokers in the EU is still high, 26% of the overall population and 29% of young Europeans aged 15–24 years smoke^1^.

The 2nd Joint Action on Tobacco Control (JATC 2), was launched in October 2021, in the wake of the 1st Joint Action on tobacco control (JATC) which ended in December 2020^2^. This second European joint project is particularly necessary, not only to give a definitive reinforcement to what has already been achieved in the first project^3^, but also to further weaken the impact of tobacco use on the health of the population and consequent healthcare costs of tobacco use in Europe.

The overall target of the JATC 2 is to strengthen the cooperation between the Member States and the European Commission within the area of tobacco control, specifically concerning enforcement and room for improvement of the Tobacco Products Directive (TPD)^4^, the Tobacco Advertising Directive (TAD)^5^ and to develop a common ground for strategies on smoke-free environments and tobacco endgame strategies. The partners of JATC 2 are also committed in the new Europe’s Beating Cancer Plan^6^ where EU Member States stand together in the fight against tobacco, and in promoting activities consistent with the objectives of the WHO Framework Convention on Tobacco Control (FCTC)^7^.

As in other Joint Actions, the intermediate and final outcomes are presented as milestones and deliverables. By the end of the project, 53 milestones and 44 deliverables will be produced, the latter available in the EU portal, on the JATC 2 website, and disseminated through different activities.

In this EU funded 36-month project, 21 countries are participating, involving 36 different institutions, and organized in 9 Work Packages (WPs). Specific activities of the Work Packages are described as follows:

*Work Package 1 – Coordination* has the objective of coordinating the overall smooth implementation of the project and ensure efficient management of the project.

*Work Package 2 – Dissemination* aims at maximizing the impact of the project by supporting the consultation with stakeholders and the dissemination of the project’s results to the target audiences.

*Work Package 3 – Evaluation of the JATC 2* evaluates the outputs and outcomes of the JATC 2 and supports the optimization of the project implementation and internal processes necessary for their achievement.

*Work Package 4 – Sustainability and Cooperation across Europe* has the objective of ensuring that the results of the Joint Action, its outputs, activities and benefits are developed and continue after the end of the project through sustainable funding and resources. Specifically, the sustainability actions are aimed at the further development of the EU cooperation on tobacco control activities through knowledge sharing and dissemination of best practices.

*Work Package 5 – EU-CEG (European Common Entry Gate) data and enhanced laboratory capacity for regulatory purposes* focuses on tobacco and related products from the point of view of their characteristics as reported into the manufacturers’ notifications through the European Common Entry Gate (EU CEG)^8^ or analyzed by independent laboratories. By acting as a focal point between end-users from Member States Competent Authorities and the European Commission, WP5 partners aim at strengthening cooperation, providing relevant tools and sustainable support to end users with regard to EU CEG data handling and product analyses, for effective enforcement of the regulations^9^.

*Work Package 6 – Enforcement of tobacco product regulation* aims at strengthening the EU Member States’ capacities in the enforcement of tobacco product regulation at the EU Member States and EU wide level through the sharing of common experiences, challenges and solutions. WP6 will establish contact with all EU authorities working with tobacco regulation to conduct a needs assessment and create a knowledge-hub network. The knowledge-hub network will consist of a platform for sharing knowledge and twice-yearly meetings for topics picked by member states.

*Work Package 7 – Health impact and regulatory implications of e-cigarettes and novel tobacco products* aims at enhancing the understanding of the properties, health impact and regulatory implications of novel tobacco products and e-cigarettes to support effective information and regulation. This will be accomplished by collecting and analyzing data to gain insight into the variation of novel tobacco products and e-cigarettes between countries. The use, abuse potential and health risks of novel tobacco products and e-cigarettes will be evaluated. In addition, adverse effects associated with the use of novel tobacco products and e-cigarettes across the EU will be collected. The results will support EU Member States’ training, capacity building and information sharing on novel tobacco products and e-cigarettes.

*Work Package 8 – Smoke-free environments and tobacco advertising, promotion, and sponsorship (TAPS) legislation in Europe* has the objective of outlining and disseminating best practices for addressing upcoming challenges to smoke-free environments in Europe (FCTC Art. 8) and to assess tobacco advertisement, promotion and sponsorship (TAPS) implementation and impact in Europe (FCTC Art. 13). WP8 will achieve this by outlining best practices and ensuring the dissemination thereof. This WP will also gather evidence in favor of smoke-free environments to identify, adapt and assess novel challenges to smoke-free environments. Further, WP8 will assess and create the framework for the expansion of smoke-free environments in Europe, including but not limited to outdoor areas and some private settings. Lastly, the WP will identify and share actions undertaken by Member States to address challenges for the application of the EU bans on cross-border and internet TAPS and develop the ‘weight of evidence’ for a new TAD.

*Work Package 9 – Best practices to develop an effective and comprehensive tobacco endgame strategy* provides tools to put forward actions in line with the ‘Tobacco-Free Generation’ goal of the Europe’s Beating Cancer Plan, of <5% of the population using tobacco by 2040 in Europe. For this purpose, the WP identifies national tobacco endgame strategies and forward-looking tobacco control policies aiming at tobacco endgame, and assesses their feasibility for the countries in the European region, taking into account different national contexts and capacities for tobacco control. The WP will also explore and exchange best practices in the development, implementation and evaluation of these strategies and policies, and synthesize evidence and identify research needs related to new policies.

The JATC 2 will contribute to the efforts to reduce the toll of tobacco-related morbidity and mortality in the European Union, through closer cooperation and a smooth circulation of relevant information on tobacco policies and strategies among the Member States. From a public health perspective, prevention, monitoring and treatment of tobacco and nicotine products use in Europe will contribute to the reduction of demand for these products, which benefits the health of the population. All this can be achieved in synergy with a comprehensive and successful planned update of the Tobacco Products Directive, the Tobacco Advertising Directive, and effective silencing of industry interference, coherent with Article 5.3 of the FCTC.

## Data Availability

Data sharing is not applicable to this article as no new data were created.
